# Molecular Profile of Barrett’s Esophagus and Gastroesophageal Reflux Disease in the Development of Translational Physiological and Pharmacological Studies

**DOI:** 10.3390/ijms21176436

**Published:** 2020-09-03

**Authors:** Edyta Korbut, Vincent T Janmaat, Mateusz Wierdak, Jerzy Hankus, Dagmara Wójcik, Marcin Surmiak, Katarzyna Magierowska, Tomasz Brzozowski, Maikel P Peppelenbosch, Marcin Magierowski

**Affiliations:** 1 Department of Physiology, Jagiellonian University Medical College, 31-531 Cracow, Poland; edyta.korbut@uj.edu.pl (E.K.); mateusz.wierdak@uj.edu.pl (M.W.); dagmara1.wojcik@uj.edu.pl (D.W.); marcin.surmiak@uj.edu.pl (M.S.); katarzyna.magierowska@uj.edu.pl (K.M.); mpbrzozo@cyf-kr.edu.pl (T.B.); 2 Department of Gastroenterology and Hepatology, Erasmus MC—University Medical Center Rotterdam, 3015 CN Rotterdam, The Netherlands; v.janmaat@erasmusmc.nl (V.T.J.); m.peppelenbosch@erasmusmc.nl (M.P.P.); 3 2nd Department of General Surgery, Jagiellonian University Medical College, 30-688 Cracow, Poland; 4 Department of Pathomorphology, Jagiellonian University Medical College, 31-531 Cracow, Poland; jerzy.hankus@uj.edu.pl; 5 Department of Internal Medicine, Jagiellonian University Medical College, 31-066 Cracow, Poland

**Keywords:** Barrett’s esophagus, esophageal epithelium, molecular profile, molecular gastrointestinal pharmacology, molecular gastrointestinal pathophysiology

## Abstract

Barrett’s esophagus (BE) is a premalignant condition caused by gastroesophageal reflux disease (GERD), where physiological squamous epithelium is replaced by columnar epithelium. Several in vivo and in vitro BE models were developed with questionable translational relevance when implemented separately. Therefore, we aimed to screen Gene Expression Omnibus 2R (GEO2R) databases to establish whether clinical BE molecular profile was comparable with animal and optimized human esophageal squamous cell lines-based in vitro models. The GEO2R tool and selected databases were used to establish human BE molecular profile. BE-specific mRNAs in human esophageal cell lines (Het-1A and EPC2) were determined after one, three and/or six-day treatment with acidified medium (pH 5.0) and/or 50 and 100 µM bile mixture (BM). Wistar rats underwent microsurgical procedures to generate esophagogastroduodenal anastomosis (EGDA) leading to BE. BE-specific genes (keratin (*KRT)1*, *KRT4*, *KRT5*, *KRT6*A, *KRT13*, *KRT14*, *KRT15*, *KRT16*, *KRT23*, KRT24, *KRT7*, *KRT8*, *KRT18*, *KRT20*, trefoil factor (*TFF*)*1*, *TFF2*, *TFF3*, villin (*VIL*)*1*, mucin (*MUC*)*2*, *MUC3A/B*, *MUC5B*, *MUC6* and *MUC13*) mRNA expression was assessed by real-time PCR. Pro/anti-inflammatory factors (interleukin (IL)-1β, IL-2, IL-4, IL-5, IL-6, IL-10, IL-12, IL-13, tumor necrosis factor α, interferon γ, granulocyte-macrophage colony-stimulating factor) serum concentration was assessed by a Luminex assay. Expression profile in vivo reflected about 45% of clinical BE with accompanied inflammatory response. Six-day treatment with 100 µM BM (pH 5.0) altered gene expression in vitro reflecting in 73% human BE profile and making this the most reliable in vitro tool taking into account two tested cell lines. Our optimized and established combined in vitro and in vivo BE models can improve further physiological and pharmacological studies testing pathomechanisms and novel therapeutic targets of this disorder.

## 1. Introduction

Barrett’s esophagus (BE) is a complex, genetically predisposed, premalignant condition of the distal esophagus characterized as replacement of the esophageal squamous epithelium into an intestinal-type columnar epithelium with a crypt-like architecture [1,2,3]. Epithelium in BE is usually composed of mucous-producing cells, which aids in the protection of the esophagus from the constant insult of acid and bile [2]. BE affects 2% of the adult population in the Western world [4,5]. It has been confirmed that chronic gastroesophageal reflux disease (GERD) is one of the most important etiological component of BE [4,5].

Importantly, BE and GERD are closely associated with a high risk of developing esophageal adenocarcinoma (EAC). EAC has a very poor prognosis with a 9–15% five-year survival rate [4]. The pattern of reflux is a significant factor that may influence the progression of BE towards advanced precancerous changes [6,7]. In contrast to the physiological esophageal epithelium, the BE development results in alternation of several individual molecular markers and signaling pathways. These changes include variety of mucins (MUC), mucin-associated trefoil factor family (TFF) peptides, and villin (VIL) [8,9,10]. However, the physiological and pathophysiological aspects still require further investigation, especially in the context of the implementation of novel non-invasive methods of treatment of this disorder.

Both BE and GERD are related to inflammation of the esophageal epithelium. Chronic inflammation in BE has been linked to DNA damage, leading to mutations and genomic instability, and altered expression of genes that are involved in cellular proliferation and programmed cell death [11]. The inflammatory response also includes increased oxidative stress, activation of several signaling pathways, and the release of inflammatory cytokines [11]. Moreover, chronic inflammation can lead to a higher rate of cellular turnover, which is typical for BE and can alter the pattern of gene expression in epithelial cells [11]. For example, an analysis of keratin (KRT), a major constituent of the esophageal epithelium, revealed significant changes from those keratins normally expressed in squamous epithelia to those expressed in columnar epithelium [8,12,13].

The diagnostic criteria for BE phenotype requires endoscopic identification of columnar mucosa and microscopic appearance of columnar epithelium with presence of goblet cells within the esophageal mucosa [3]. The basic therapeutic options for patients with BE include pharmacological treatment with proton pump inhibitors or endoscopic procedures with surgical resection, and chemo- and radiotherapy [14]. However, the evidence from randomized controlled trials shows that pharmacological and surgical therapies do not completely prevent or eliminate BE and existing dysplasia [14]. Therefore, the development of novel or alternative pharmacological therapeutic interventions seems to be justified, also taking into account the recently published evidence [5]. Current advances in the understanding of the complex molecular mechanisms of BE development, which came from experimental models of BE, include overexpression of cyclooxygenase-2 (COX-2) [15,16], epidermal growth factor (EGF) [17,18,19], or mitogen-activated protein kinase (MAPK) and the protein kinase phosphorylation (PI3K) pathways [20,21], as well as increased secretion of gastrin due to achlorhydria as a complication of prolonged proton pomp inhibitors (PPI) therapy [22]. However, many ongoing controversies and challenges and potential mediators responsible for the development of BE still remain unsolved. Additionally, the conversion process of normal squamous epithelium towards Barrett’s metaplasia is difficult to monitor directly under clinical conditions [23]. Thus, over the last few years, several experimental models using various types of cell cultures and animal models have been published to investigate the mechanisms of bile and/or acid exposure in BE pathogenesis [1,23]. However, the relevance of each of the models implemented separately is considered to be questionable.

Therefore, this study was designed to establish relevant experimental models for further studies emphasizing the effectiveness of possible protective treatment of BE esophageal metaplasia with pharmacological agents. Thus, we selected appropriate translational molecular markers such as *KRT*, *MUC*, *TFF* and *VIL* genes to compare expression profiles in clinical biopsies derived from BE patients with the animal surgical model and an in vitro model of BE involving two human-derived primary immortalized esophageal cell lines. We put special emphasis on the optimization of this in vitro model with the aim to reflect the molecular events observed clinically as closely as possible.

## 2. Results

### 2.1. Analysis of BE Expression Profile for Selected Genes in Human Biopsies Based on GSE Datasets

Expression of mRNA for squamous epithelium-specific genes such as *KRT1*, *KRT4*, *KRT5*, *KRT6A-C*, *KRT13*, *KRT14*, *KRT15*, *KRT16*, *KRT23*, KRT24 was significantly decreased in human Barrett’s esophagus biopsies as compared with expression of these specific genes in samples collected from normal squamous epithelium and observed in at least one out of three analyzed databases (*p* < 0.05, Table 1). Expression of mRNA for columnar and intestinal epithelium-specific genes such as *KRT7*, *KRT8*, *KRT18*, *KRT20*, *TFF1*, *TFF2*, *TFF3*, *VIL1*, *MUC2*, *MUC3A/B*, *MUC5B*, *MUC6*, *MUC13* was significantly upregulated in human BE biopsies when compared with gene expression in samples collected from normal squamous epithelium in at least one out of three analyzed databases (*p* < 0.05, Table 1). Genes such as *KRT10*, *KRT17*, *KRT19*, *MUC1*, *MUC5ac*, *MUC12*, *MUC15*, *MUC17* and *MUC21* did not fulfil our selection criteria and were not interpreted (Table 1).

### 2.2. The Effect of Exposition to Various Bile Mixture (BM) Concentrations and pH Values on the Viability of Esophageal Epithelial Cell Lines

**Figure 1:**
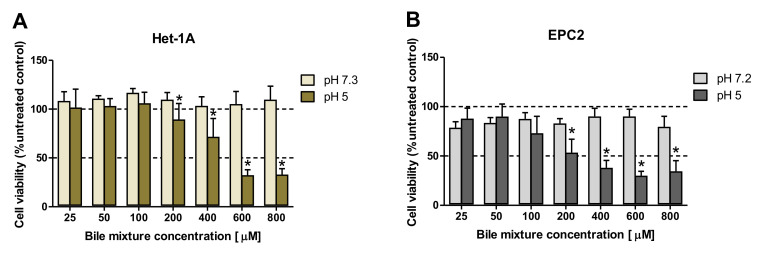
The effect of exposure to various concentrations of bile mixture (BM) and different pH on the viability of esophageal epithelial cell lines. Het-1A (**A**) and EPC2 (**B**) cells were treated for 30 min with BM at concentrations ranging from 0 to 800 μM. After 24 h, MTT assay was used to determine cell viability. Significant changes (*p* < 0.05) in cell viability after BM treatment at pH 5.0 as compared to the treatment with BM in regular medium were indicated by asterisk (*).

To determine the effect of the exposure to BM on cell viability, cells were incubated for 30 min with various BM concentrations (0–800 μM) in regular or low pH (pH 5.0) cell culture medium (Figure 1A,B). Figure 1A,B show no significant changes in cell viability when BM (0–800 μM) was applied in regular medium in both cell lines as compared with control cells not exposed to BM. In contrast, when Het-1A and EPC2 cells were incubated with BM in the concentrations ≥200 µM and at low pH, a dose-dependent and significant decrease in cell viability was observed in comparison to the respective doses of BM applied in regular medium (*p* < 0.05, Figure 1A,B).

### 2.3. Optimization of the Experimental Procedure Duration

**Figure 2:**
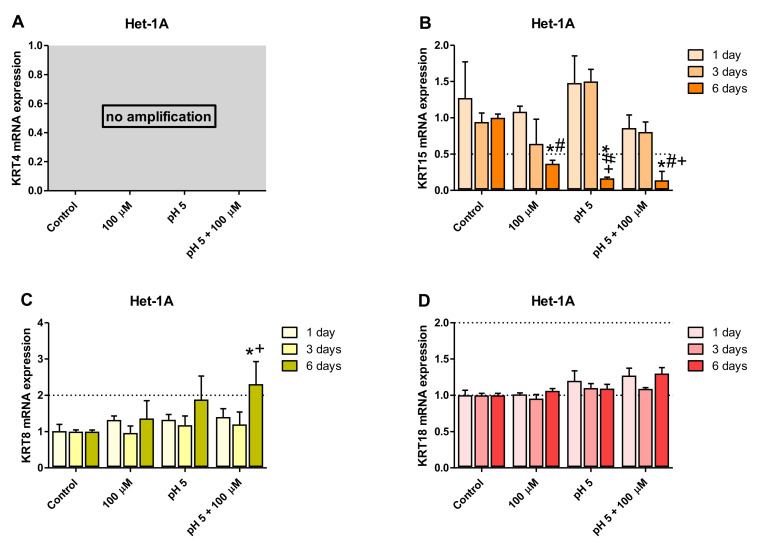
The effect of treatment with bile mixture (BM), acidified medium alone or acidic BM for one, three and six consecutive days on *KRT4*, *15*, *8* and *18* mRNA expression in Het-1A cell line. Het-1A cell line underwent 30 min of daily incubation with BM at concentration of 100 µM, acidified medium to pH 5.0 alone or BM (100 µM) in acidified medium (pH 5.0) for one, three or six consecutive days and expression of *KRT4*, *KRT15*, *KRT8* and *KRT18* mRNA was analyzed by real-time PCR (**A**–**D**). PCR reaction was performed in duplicates and quantified using *ACTB*/*GAPDH* as reference genes. Data from three independent experiments are shown as the mean ± SEM. An asterisk (*) indicates a significant change as compared with untreated control cells (*p* < 0.05). Significant change (*p* < 0.05) in gene expression as compared with one day exposure to respective treatment regime is indicated by a hash (#). A cross (+) indicates a significant change as compared with cells after three days of treatment for respective treatment regime (*p* < 0.05).

**Figure 3:**
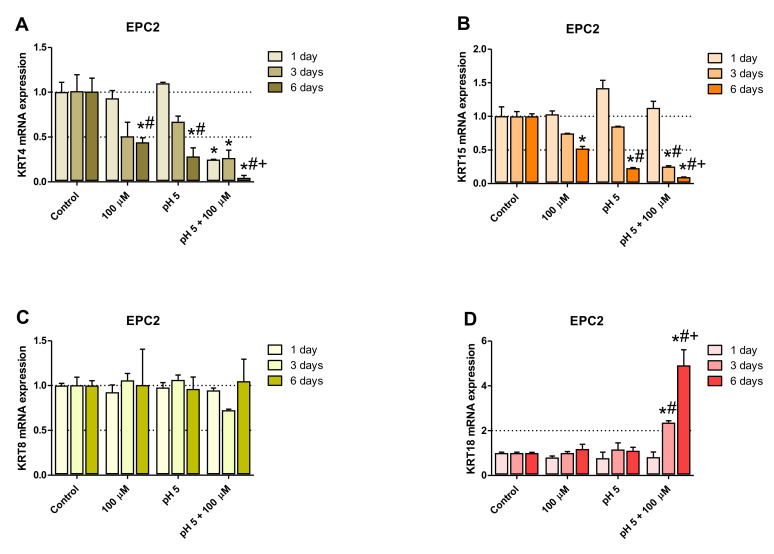
The effect of treatment with bile mixture (BM), acidified medium alone or acidic BM for one, three and six consecutive days on *KRT4*, *15*, *8* and *18* mRNA expression in EPC2 cell line. EPC2 cell line underwent 30 min of daily incubation with BM at concentration of 100 µM, acidified medium to pH 5.0 alone or BM (100 µM) in acidified medium (pH 5.0) for one, three or six consecutive days and expression of *KRT4*, *KRT15*, *KRT 8* and *KRT18* mRNA was analyzed by real-time PCR (**A**–**D**). PCR reaction was performed in duplicates and quantified using *ACTB/GAPDH* as reference genes. Data from three independent experiments are shown as the mean ± SEM. An asterisk (*) indicates a significant change as compared with untreated control cells (*p* < 0.05). Significant change (*p* < 0.05) in gene expression as compared with one day exposure for respective treatment regime is indicated by hash (#). A cross (+) indicates a significant change as compared with cells after three days of treatment (*p* < 0.05).

To identify the optimal duration of acidic BM treatment in the establishment of an in vitro model of BE-type molecular profile development, based on the analysis of GSE datasets, two squamous (*KRT4*, *KRT15*) and two columnar (*KRT8*, *KRT18*) epithelium-specific genes were randomly selected. For this purpose, Het-1A (Figure 2A–D) and EPC2 (Figure 3A–D) cell lines were daily exposed for 30 min to acidified medium (pH 5.0) and/or 100 µM of bile mixture (BM) for one, three or six consecutive days. The concentration of 100 µM of BM (pH 5.0) was selected based on previous experiments documenting that this concentration was the highest concentration at which the cell viability was not significantly affected (Figure 1A,B).

No amplification of *KRT4* gene was observed in Het-1A cell line (Figure 2A) but Figure 3A shows that at 100 µM BM in acidified medium, a significant downregulation of the expression of squamous epithelium-specific *KRT4* mRNA in EPC2 cells was observed after one, three and six days of treatment, in comparison to untreated control cells (*p* < 0.05). Moreover, *KRT4* mRNA level in EPC2 cells was significantly decreased after six days in comparison to one and three days of treatment (*p* < 0.05; Figure 3A). Additionally, the low pH and 100 µM BM applied separately to EPC2 cells for six but not three days significantly inhibited *KRT4* mRNA expression in comparison to untreated control cells and to respective experimental group after one day of treatment (*p* < 0.05; Figure 3A).

Figure 2B and Figure 3B show significant downregulation of *KRT15* mRNA expression in both cell lines after six days as compared to untreated control cells and to one and three days of treatment with BM at pH 5.0 (*p* < 0.05). Additionally, in Het-1A cells low pH alone decreased levels of *KRT15* mRNA after six days in comparison to untreated control cells and to cells after one and three days of treatment (*p* < 0.05; Figure 2B). Moreover, we have noticed that 100 µM BM applied at regular medium to Het-1A cells for six days significantly downregulated *KRT15* mRNA expression in comparison to untreated control cells and to cells after one day of treatment (*p* < 0.05; Figure 2B). In EPC2 cells low pH alone inhibited *KRT15* mRNA expression after 6 days, in comparison to untreated control cells and to cells after one day of treatment (*p* < 0.05; Figure 3B). BM applied in concentration 100 µM (regular pH 7.2) for six days significantly decreased *KRT15* mRNA level in EPC2 cells in comparison to respective untreated control cells (*p* < 0.05; Figure 3B).

Our time sequence determination revealed that a significant upregulation of *KRT8* mRNA was observed exclusively in Het-1A but not in EPC2 cells after six days as compared with one and three days of treatments or with untreated control cells (*p* < 0.05; Figure 2C and Figure 3C). *KRT18* mRNA was significantly upregulated in EPC2 but not in Het-1A cell line after three and six days as compared with one day of treatment or with untreated control cells (*p* < 0.05; Figure 2D and Figure 3D).

To summarize, two out of three and three out of four genes from the BE molecular profile, Het-1A and EPC2, respectively, show a significantly higher fold change after six days of acidic BM treatment, compared to one or three days of treatment. As the fold change in expression between BE and squamous epithelium is most resembled by the model using six days of acidic BM treatment, this condition was selected for further study.

### 2.4. Squamous and Columnar Epithelium-Specific Genes Expression in In Vitro Model

**Figure 4:**
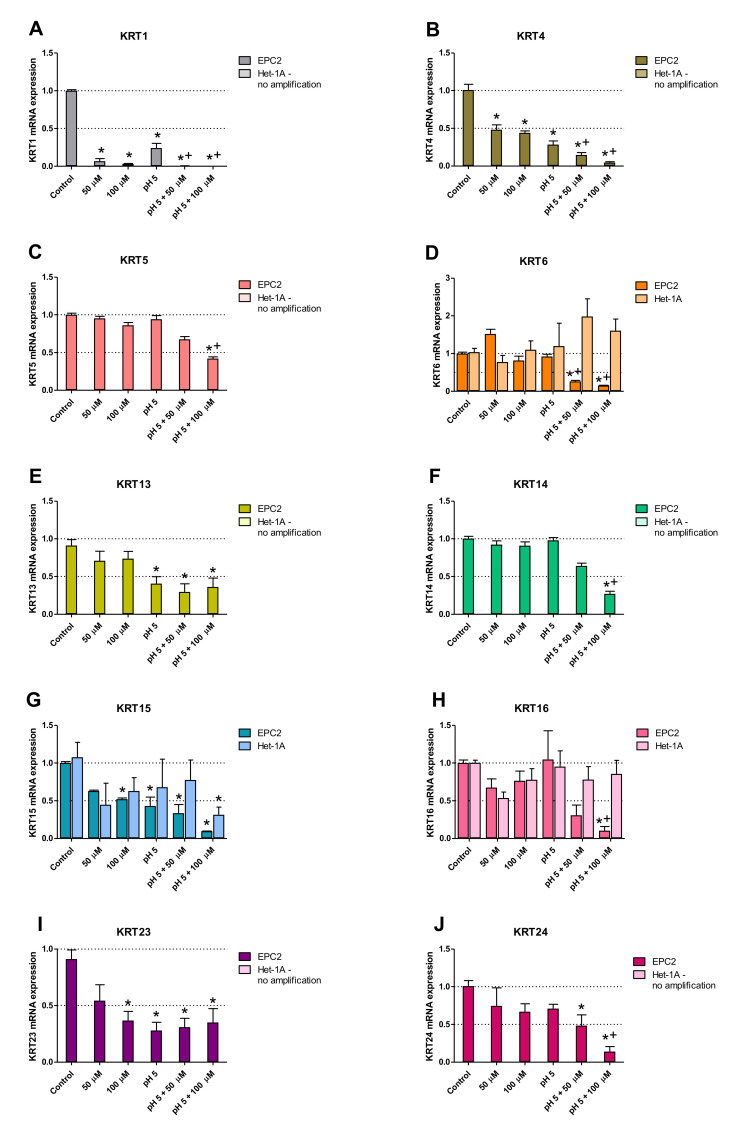
Squamous epithelium-specific mRNA expression upon incubation of Het-1A and EPC2 cells with bile mixture (BM) at pH 5.0. Het-1A and EPC2 cell lines were incubated with BM (50 µM and 100 µM) in acidified medium (pH 5.0) or regular medium for six consecutive days and squamous epithelium-specific mRNA expression was analyzed by real-time PCR (**A**–**J**). PCR reaction was performed in duplicates and quantified using *ACTB/GAPDH* as reference genes. Data from three independent experiments are shown as the mean ± SEM. Significant change in gene expression after six days of treatment as compared with untreated control cells is indicated by an asterisk (*) (*p* < 0.05). A cross (+) indicates a significant change as compared with cells incubated with pH 5.0 alone (*p* < 0.05).

Figure 4A–J show changes in mRNA expression for squamous epithelium-specific genes, selected based on Table 1 analysis, as characteristic for human BE biopsies, determined in Het-1A and EPC2 cells treated for 30 min per day with 0 µM, 50 µM, and 100 µM BM at either pH7.2/3 or pH 5.0, for six consecutive days. Incubation of EPC2 cells with 100 µM BM at pH 5.0 resulted in a significant decrease in expression of all investigated squamous epithelium-specific *KRT* (*KRT1*, *KRT4*, *KRT5*, *KRT6*, *KRT13*, *KRT14*, *KRT15*, *KRT16*, *KRT23, KRT24*) genes as compared to untreated control cells (*p* < 0.05; Figure 4A–J). Significant downregulation of *KRT1* (4A), *KRT4* (4B), *KRT5* (4C), *KRT6* (4D), *KRT14* (4F), *KRT16* (4H) and KRT24 (4J) mRNA as compared to EPC2 cells incubated with low pH alone was observed (*p* < 0.05). Incubation of EPC2 cells with lower concentration of BM (50 µM) at pH 5.0 significantly downregulated mRNA expression of *KRT1* (4A), *KRT4* (4B), *KRT6* (4D), *KRT13* (4E), *KRT15* (4G) *KRT23* (4I) and *KRT24* (4J) in comparison to untreated control cells (*p* < 0.05). Exposure of EPC2 cells to 100 µM of BM at regular medium resulted in a significant decrease in *KRT1* (4A), *KRT4* (4B), *KRT15* (4G) and *KRT23* (4I) mRNA levels as compared to untreated control cells (*p* < 0.05). In turn, 50 µM of BM applied alone inhibited mRNA expression of *KRT1* (4A) and *KRT4* (4B) in comparison to untreated control EPC2 cells (*p* < 0.05). The mRNA expression of *KRT1* (4A), *KRT4* (4B), *KRT13* (4E), *KRT15* (4G) and *KRT23* (4I) in EPC2 cells cultured in acidified medium (pH 5.0) without BM was significantly inhibited as compared to untreated control cells (*p* < 0.05). In Het-1A cells, no mRNA amplification of *KRT1* (4A), *KRT4* (4B), *KRT5* (4C), *KRT13* (4E), *KRT14* (4F) *KRT23* (4I) and KRT24 (4J) and no changes in mRNA expression of *KRT 6* (4D) and *KRT16* (4H) was observed in all experimental groups. Only *KRT15* mRNA expression was significantly downregulated in Het-1A cells after the treatment with BM (100 µM) at pH 5.0, as compared to untreated control cells (*p* < 0.05; Figure 4G).

**Figure 5:**
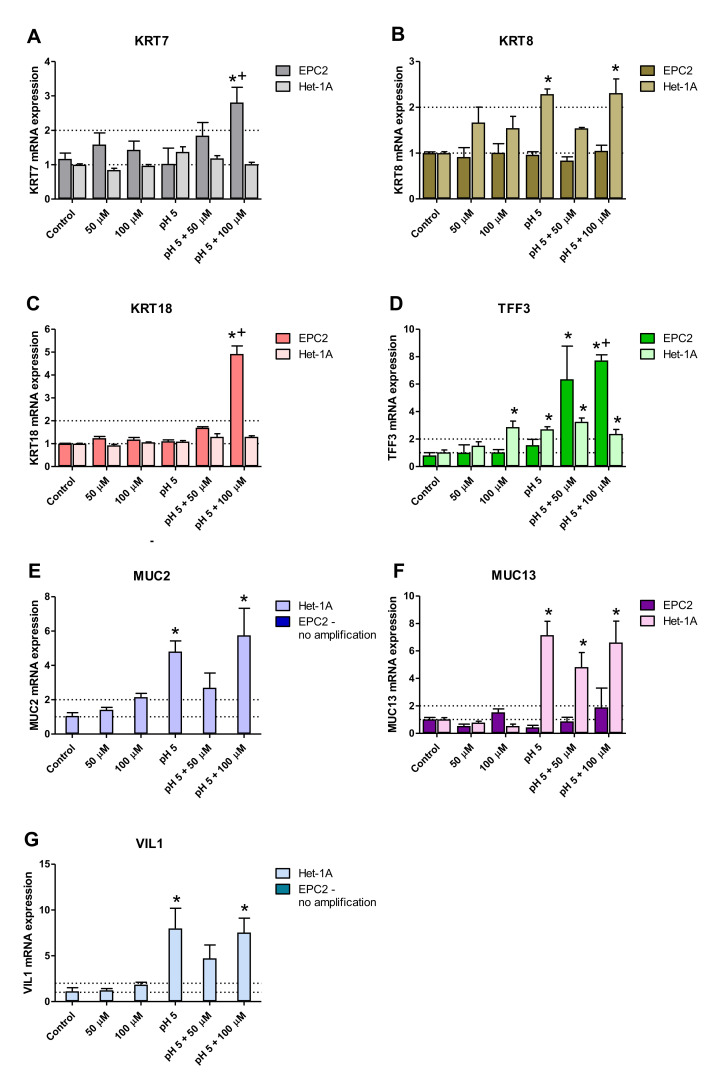
Columnar epithelium-specific mRNA expression upon incubation of Het-1A and EPC2 cells with bile mixture (BM) at pH 5.0. Het-1A and EPC2 cell lines were incubated with BM (50 µM and 100 µM) in acidified medium (pH 5.0) or regular medium for six consecutive days and squamous epithelium-specific mRNA expression was analyzed by real-time PCR (**A**–**G**). PCR reaction was performed in duplicates and quantified using *ACTB/GAPDH* as reference genes. Data from representative three independent experiments are shown as the mean ± SEM. Significant change in gene expression after six days of treatment as compared with untreated control cells is indicated by an asterisk (*) (*p* < 0.05). A cross (+) indicates a significant change as compared with cells incubated with pH 5.0 alone (*p* < 0.05).

Figure 5A–G show mRNA expression of columnar epithelium-specific genes, selected based on Table 1 analysis, altered in patients with BE, and determined in Het-1A and EPC2 cells. In EPC2 cells treated with 100 µm BM at pH 5.0, significant upregulation of *KRT7* (5A), *KRT18* (5C) and *TFF3* (5D) mRNA expression in comparison to untreated control cells and cells treated with low pH alone was observed (*p* < 0.05). In contrast, exposure to 50 µM BM at pH 5.0 significantly elevated only *TFF3* mRNA expression in EPC2 cells (*p* < 0.05; Figure 5D). When BM (100 µm) at pH 5.0 was co-incubated with Het-1A cells, the upregulation of *KRT8* (5B), *TFF3* (5D), *MUC2* (5E), *MUC13* (5F) and *VIL1* (5G) mRNA was detected as compared to untreated control cells (*p* < 0.05). In turn, BM applied at the concentration of 50 µM at pH 5.0 significantly elevated only *TFF3* (5D) and *MUC13* (5F) mRNA expression over the mRNA expression levels obtained in untreated control Het-1A cells (*p* < 0.05). Likewise, BM (100 µM) applied at regular medium significantly increased mRNA expression of *TFF3* as compared to untreated control Het1A cells (*p* < 0.05; Figure 5D). In Het-1A cells cultured in acidified medium (pH 5.0) without BM significant upregulation of *KRT8* (5B), *TFF3* (5D), *MUC2* (5E), *MUC13* (5F) and *VIL1* (5G) mRNA was determined as compared to untreated control cells (*p* < 0.05). No amplification was observed for either *KRT20*, *TFF1*, *TFF2*, *MUC6*, *MUC5B* and MUC3A/B mRNA in all experimental groups for both cell lines (data not shown).

### 2.5. Morphology of Esophageal Mucosa, Gastroesophageal Junction (GEJ), and Gastric Cardia in Rats with Esophagogastroduodenal Anastomosis (EGDA)

Table 2 shows that in rats with 10 weeks of EGDA, the lesion score assessed macroscopically reached three in 70% of cases (7 out of 10 animals). In 20% of rats, disease progression reached score four and only 10% of animals reached lesion score two (Table 2).

**Figure 6:**
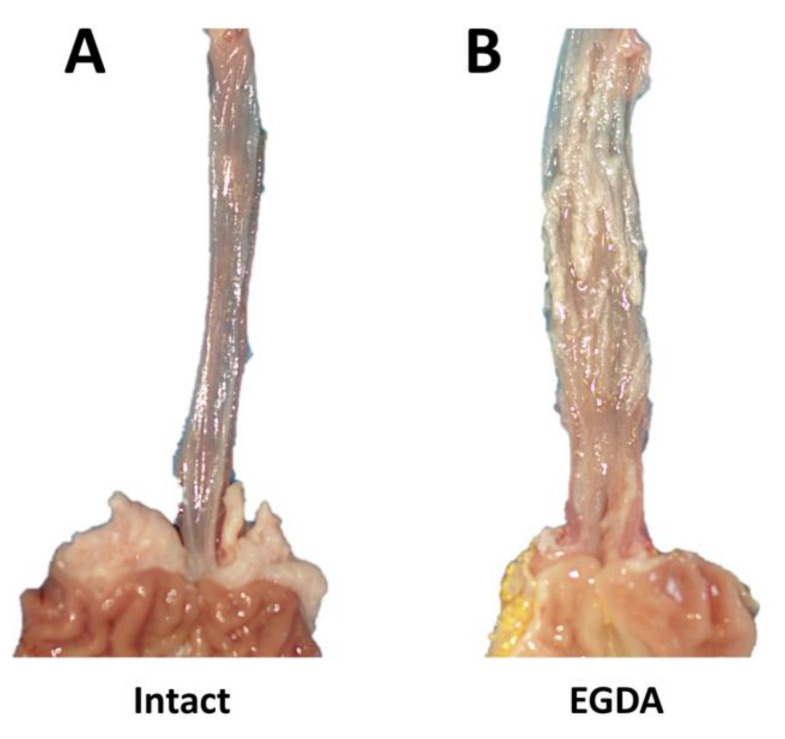
Macroscopic appearance of esophageal mucosa, gastroesophageal junction and gastric cardia in representative rats without (Intact, **A**) or with esophagogastroduodenal anastomosis (EGDA, **B**).

Figure 6 shows the macroscopic appearance of esophageal mucosa, GEJ, and gastric cardia of representative rats with or without EGDA (Figure 6A,B, respectively). After exposure to chronic reflux due to EGDA, thickening of esophageal wall with ulceration and papillomatosis of the esophageal mucosa surface was observed (Figure 6B).

Table 3 shows that all 10 animals with EGDA developed hyperplasia of squamous epithelium and fibrosis of the lamina propria at 10 weeks after surgery. In 80% of the rats, esophagitis with ulceration was observed (Table 3). Barrett’s metaplasia was present in 60% of the rats (Table 3).

**Figure 7:**
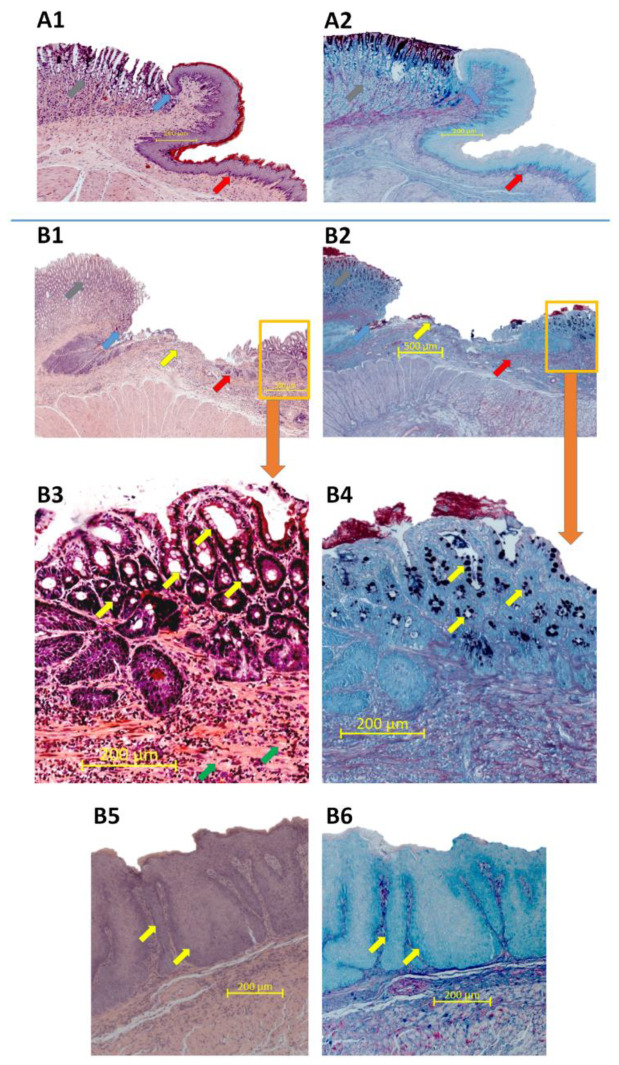
Microscopic appearance of esophageal mucosa, gastroesophageal junction (GEJ) and gastric cardia stained with haematoxylin/eosin (H&E) or alcian blue/periodic acid-Schiff (AB/PAS) in representative rats without (**A1**,**A2**) or with an esophagogastroduodenal anastomosis (**B1**–**B6**). Grey arrow points out gastric mucosa, blue arrow points out GEJ, red arrow indicates esophageal mucosa, yellow arrow indicates esophageal ulceration, orange frame shows Barrett’s-like lesions (**A1**,**A2**,**B1**,**B2**). (**B3**,**B4**) show high resolution images of Barrett’s-like lesions where yellow arrows indicate goblet cells and green arrows indicate fibrosis. Yellow arrows indicate epithelial hyperplasia (**B5**,**B6**).

Figure 7A1,A2 shows the microscopic appearance of the esophageal mucosa, GEJ, and gastric mucosa obtained from representative intact rats. The typical morphology manifestation of hyperplasia, fibrosis or inflammation of experimental BE was not observed in esophageal epithelium and submucosa attached to the GEJ of intact rats (Figure 7A1) and the AB/PAS staining did not show any pathological changes within the tissue of these rats (Figure 7A2). In contrast, Figure 7B1,B2 shows that in rats with EGDA, the GEJ architecture is altered. Esophageal mucosa is characterized by evident ulceration and fibrosis as demonstrated in the high resolution image (Figure 7B3). Moreover, Barrett’s-like lesions metaplasia with presence of AB-positive goblet cells is observed proximally from the GEJ (Figure 7B3,B4). Figure 7B5,B6 presents photomicrographs of esophageal tissue section collected separately from the same rate, as shown in Figure 7B3,B4. In animals with EGDA, the esophageal squamous mucosa shows pronounced epithelial hyperplasia (Figure 7B5,B6).

### 2.6. Alterations in Serum Content of Pro- and Anti-Inflammatory Cytokines in Rats with EGDA

**Figure 8:**
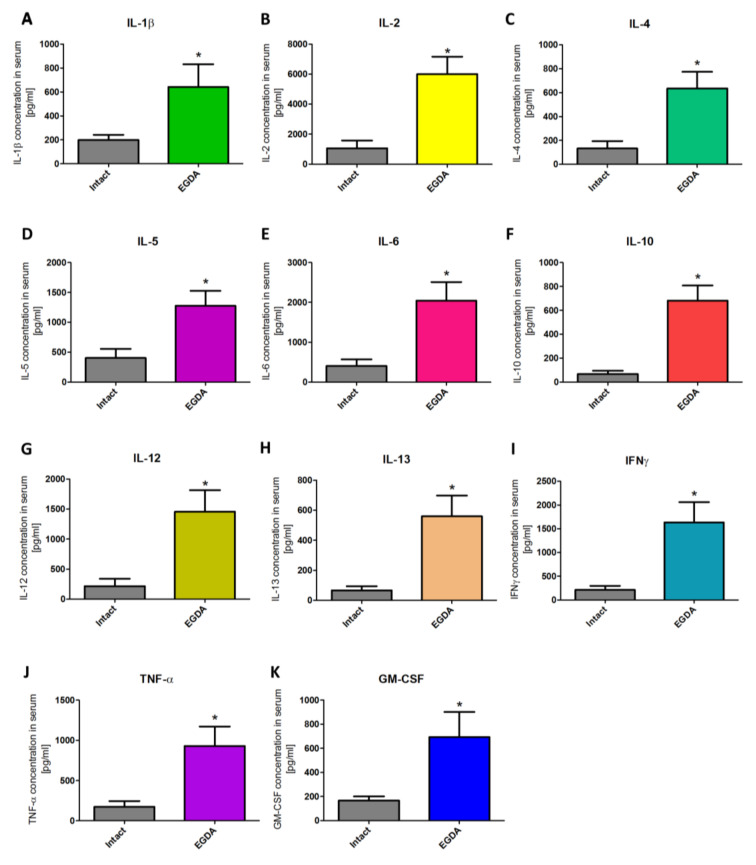
Serum concentration of interleukin (IL)-1β (**A**), IL-2 (**B**), IL-4 (**C**), IL-5 (**D**), IL-6 (**E**), IL-10 (**F**), IL-12 (**G**), IL-13 (**H**), interferon (IFN)-γ (**I**), tumor necrosis factor (TNF)-α (**J**), and granulocyte-macrophage colony-stimulating factor GM-CSF (**K**) in rats without (intact) and with esophagogastroduodenal anastomosis (EGDA). Results are mean ± SEM of five samples per each experimental group. Asterisk (*) indicates a significant change as compared with respective values obtained in rats without EGDA (*p* < 0.05).

Figure 8A–K shows that serum contents of interleukin (IL)-1β (10A), IL-2 (10B), IL-4 (10C), IL-5 (10D), IL-6 (10E), IL-10 (10F), IL-12 (10G), IL-13 (10H), interferon (IFN)-γ (10I), tumor necrosis factor (TNF)-α (10J), and granulocyte-macrophage colony-stimulating factor (GM-CSF) (10K), respectively, were significantly increased in rats with EGDA as compared with intact animals (*p* < 0.05).

### 2.7. Squamous and Columnar Epithelium-Specific mRNA Expression in Esophageal Mucosa of Rats with EGDA

**Figure 9:**
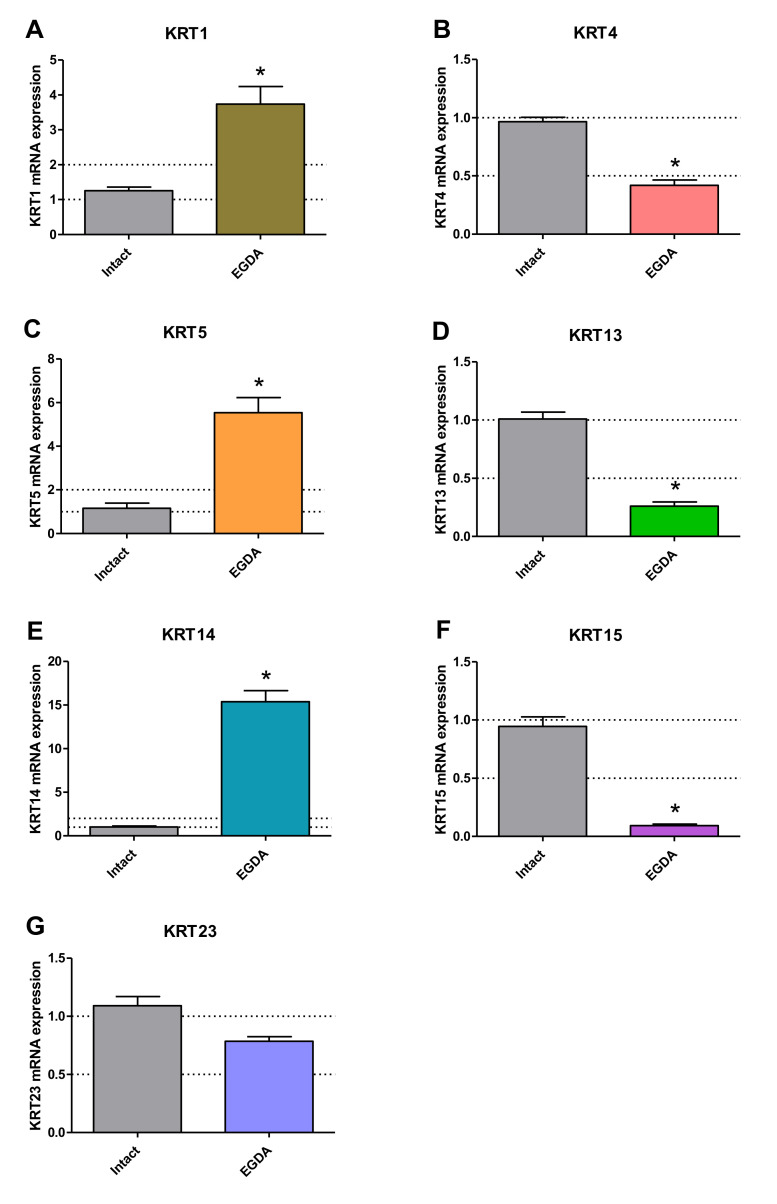
Expression of mRNA for squamous epithelium-specific genes in rats without (intact) and with esophagogastroduodenal anastomosis (EGDA). Results are expressed as mRNA expression of squamous epithelium-specific genes (**A**–**G**) normalized to *ACTB/GAPDH* expression and are mean ± SEM for *n* = 5 samples per each experimental group. Asterisk (*) indicates a significant change as compared with respective values obtained in rats without EGDA (*p* < 0.05).

Figure 9 shows expression of squamous epithelium-specific (A–G) genes in esophageal mucosa of rats with EGDA. In esophageal mucosa of rats with EGDA, *KRT4* (9A), *KRT13* (9D) and *KRT15* (9F) mRNA expression was significant downregulated in line with in vitro model and *KRT1* (9A), *KRT5* (9C) and *KRT14* (9E) mRNA fold changes were significantly increased not in line with in vitro model as compared to intact rats (*p* < 0.05). EGDA did not significantly affect mRNA expression of *KRT23* (9G).

**Figure 10:**
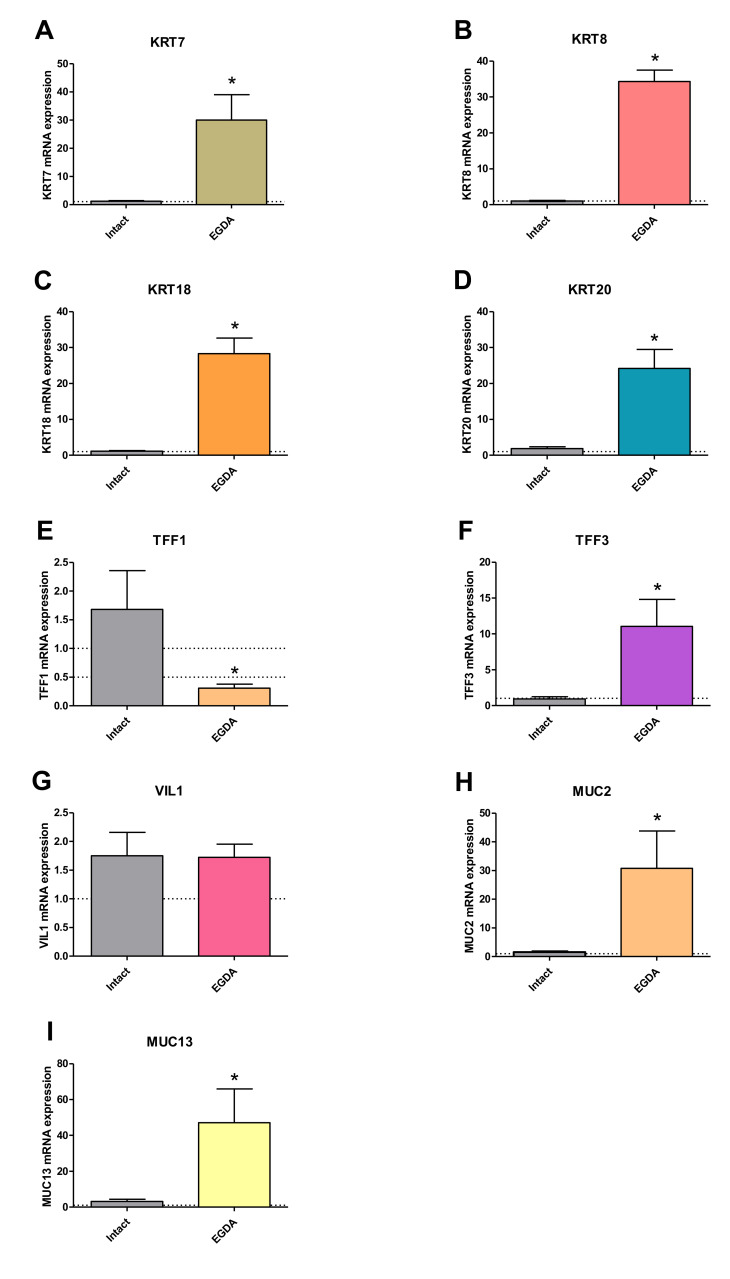
Expression of mRNA for columnar epithelium-specific genes in rats without (intact) and with esophagogastroduodenal anastomosis (EGDA). The results are expressed as mRNA expression of columnar epithelium-specific genes (**A**–**I**) normalized to *ACTB/GAPDH* expression and are the mean ± SEM for *n* = 5 Barrett’s-like samples per experimental group. Asterisk (*) indicates a significant change as compared with respective values obtained in rats without EGDA (*p* < 0.05).

Figure 10 shows that the mRNA expression of columnar epithelium-specific genes *KRT7* (10A), *KRT8* (10B), *KRT18* (10C), *KRT20* (10D), *TFF3* (10F), *MUC2* (10H) and *MUC13* (10I) was significantly upregulated in rats with EGDA in comparison to intact rats (*p* < 0.05). Only *TFF1* (10E) mRNA was downregulated after EGDA. EGDA did not lead to any significant changes in *VIL1* (10G) mRNA expression. No amplification was observed for *KRT6*, *KRT16*, *KRT24*, *TFF2*, *MUC3A*, *MUC5B* and *MUC6* mRNA in rats (data not shown).

## 3. Discussion

Human BE is a pathological condition associated with longstanding GERD and is defined as metaplasia of the flat, layered esophageal squamous epithelium into a tall intestinal columnar epithelial cells [23,25]. It is important to highlight that Barrett’s metaplasia may originate from GEJ stem cells [26,27].

In our study, we have aimed to establish an appropriate experimental model that enables the evaluation of the effectiveness of novel pharmacological tools in the pathophysiology of Barrett’s metaplasia development at the microscopic, systemic, and especially molecular levels. For this purpose, we have chosen three datasets GSE13083 [8], GSE34619 [9] and GSE1420 [24] from Gene Expression Omnibus (GEO) and applied them to the GEO2R online tool to select the commonly expressed genes in BE epithelium. We observed significant downregulation in mRNA expression for squamous epithelium-specific genes such as *KRT1*, *KRT4*, *KRT5*, *KRT6A-C*, *KRT13*, *KRT14*, *KRT15*, *KRT16*, *KRT23* and *KRT24* in human BE biopsies as compared with samples collected from normal squamous esophageal epithelium. In turn, expression of mRNA for columnar and intestinal epithelium-specific genes such as *KRT7*, *KRT8*, *KRT18*, *KRT20*, *TFF1*, *TFF2*, *TFF3*, *VIL1*, *MUC2*, MUC3A/B, *MUC5B*, *MUC6 and MUC13* was significantly upregulated. We assume that the alterations in the mRNA expression of the abovementioned specific genes reflect the development of metaplasia within the epithelium at the molecular level.

There have been several attempts to develop experimental in vivo models of GERD leading to BE and/or EAC, which attempt to mimic the clinical course of this disorder. The most widely described model in literature is the surgical animal model with rats [28]. Attwood et al. divided existing reflux models into three categories depending on the production of esophagitis alone (rat pyloric ligation, Wendel esophagogastroplasty, or external esophageal perfusion), esophagitis and BE but not EAC (total gastrectomy or mucosal excision with hiatal hernia creation), and esophagitis, BE, and EAC (esophagojejunostomy, esophagoduodenal anastomosis, or esophagogastroduodenal anastomosis) [29]. Interestingly, Quante et al. demonstrated that genetically modified mice overexpressing IL-1β also develop Barrett’s-like lesions [26].

We have selected and implemented the well-known surgical rat model based on generation of an appropriate anastomosis according to the method described previously by Nishijima et al. [30]. Microscopic and histological analysis confirmed BE metaplasia with the presence of goblet cells in 60% of rats with 10 weeks of EGDA.

Additionally, we have demonstrated an evident increase in the expression of pro/anti-inflammatory cytokines in rats with experimental gastroduodenoesophageal reflux. This is corroborative with previous findings that find that chronic inflammation of esophageal mucosa may occur as the secondary consequence of multiple exposures of esophageal structure to the acidic and alkaline content. Thus, there is no doubt that this gastroduodenal content may represent a common risk factor in the BE pathogenesis and its further progression [7,15,30,31,32].

Moreover, based on the analysis of the BE expression profile in human biopsies, we sought to identify alterations in mRNA expression of selected genes including squamous epithelium-specific (*KRT1*, *KRT4*, *KRT5*, *KRT6*, *KRT13*, *KRT14*, *KRT15*, *KRT16*, *KRT23, KRT24*) and columnar epithelium-specific (*KRT7*, *KRT8*, *KRT18*, *KRT20*) keratins together with secretory (*MUC2*, *MUC5B*, *MUC6*) and epithelial membrane-bound (*MUC3A/B*) mucins, trefoil factor family (*TFF1*, *TFF2*, *TFF3*) and villin (*VIL1*) genes in the esophageal mucosa of rats with EGDA in comparison to intact rats without EGDA. We found that expression of squamous epithelium-specific *KRT4*, *KRT13* and *KRT15* mRNA was significantly downregulated in esophageal mucosa of rats with EGDA. These findings seem to closely correlate with changes in mRNA expression as demonstrated in mucosal biopsies collected from patients with BE. In addition, among investigated columnar epithelium-specific genes *KRT7*, *KRT8*, *KRT18*, *KRT20*, *TFF3*, *MUC2* and *MUC13* mRNA expression was significantly upregulated reflecting changes observed in BE patients. In contrary, *KRT1*, *KRT5*, *KRT14*, *TFF1* and *VIL1* mRNA expression was increased in animal biopsies, which is not in line with the expression of these genes observed in human BE biopsies. This phenomenon can be explained by species-specific discrepancy between humans and rodents, especially taking into account that *KRT1, KRT5* and *KRT14* are expressed in human squamous epithelium. The direct translational character of the scientific data derived from animal studies related to BE and compared with human BE can be doubtful when considering e.g., the variability in the structure and physiology between the rodent and human esophagus (Table 4) [33,34].

The data accumulated in our study may support the notion proposed by Attwood et al. that results from animal models cannot be always translated to clinical settings [27]. Thus, if the results are achieved without the solid pathology background, experimental as well as molecular evidences, the results of subsequent work must be interpreted carefully [29]. Therefore, there is a great need for alternative methods that will be more available, will not depend on the presence of BE patients, and strive to mimic the human in vivo microenvironments in an in vitro setting [35]. For instance, Bus et al. reviewed a large variety of in vitro models and incubation conditions for studying BE development [1]. In their in vitro studies, bile salts at either a low or neutral pH were required to induce expression of BE-specific factors [1]. Moreover, they proposed that the esophageal squamous epithelium cell lines, such as the Het-1A cells, appear to be the most appropriate models for studying BE pathogenesis [1]. On the contrary, according to Underwood et al., Het-1A cell line does not possess the characteristics of normal esophageal squamous cells and should be studied with caution in translator research on BE [36]. Thus, in addition to the commonly used Het-1A cell line, we have implemented human esophageal keratinocytes EPC2 cell line to investigate their molecular response to acid and/or BM exposures.

Since the major constituent of the esophageal epithelium are the keratins [35,37], we chose them as a focal point to investigate the molecular pattern of Barrett’s metaplasia and to identify the optimal duration of acid and/or BM treatment to establish an in vitro model of BE development. Based on cell viability analysis we have selected BM at the concentration of 100 µM applied in medium adjusted to pH 5.0 as the highest concentration, which did not affect esophageal cell lines survival. We have further assessed possible time-dependent alterations in the expression of two squamous (*KRT4*, *KRT15*) and two columnar (*KRT8*, *KRT18*) epithelium-specific *KRT* genes in Het-1A and EPC2 cell lines after exposing the cells for 30 min per day to desired BM (100 µM) at pH 5.0 for one, three and six consecutive days. In the majority of cases, investigated *KRT* genes revealed the most efficient changes in mRNA expression reflecting these observed in human biopsies only when treatment was repeated for six consecutive days in contrast to those recorded at one or three days. Thus, we conclude that six days of treatment seems to be the most optimal for both tested cell-lines to induce a specific BE molecular pattern and these conditions have been chosen for further analysis of a broader spectrum of genes. Interestingly, for EPC2 cells three days of treatments were sufficient to induce this molecular pattern. Thus, to determine whether and how low pH and/or BM exposure can affect mRNA expression of squamous and columnar epithelium-specific genes observed in humans (Table 2), both Het-1A and EPC2 cells were exposed for 30 min daily for six consecutive days with BM (50 µM and 100 µM) at pH 5.0, or with BM (50 µM and 100 µM) and medium adjusted to pH 5.0 applied separately. We found that in EPC2 and Het-1A cells, BM at the concentration 50 µM at pH 5.0, as well as BM and acidified medium (pH 5.0) applied separately were less effective in induction of gene expression changes characteristic for BE patients in comparison to the experiments in which BM in higher concentration of 100 µM has been applied at pH 5.0. This clearly indicates that changes in the specific gene expression are dependent on bile concentration and acidic environment. Moreover, we observed that incubation of EPC2 cells with 100 µM of BM at pH 5.0 downregulated mRNA expression of all investigated squamous epithelium-specific KRT genes as compared to untreated control cells. Interestingly, in Het-1A cells only *KRT15* mRNA expression was significantly downregulated. This is in accordance with observations by Mari et al. [38], who claimed that Het-1A cells lacked the expression of majority of squamous epithelium-specific KRT genes, confirming that this cell line has an incomplete squamous phenotype. In addition, in our study, more columnar epithelium-specific genes were upregulated in Het-1A in comparison to EPC2 under optimized experimental conditions. For instance, when Het-1A cells were treated with 100 µM BM at pH 5.0, the expression of columnar epithelium-specific *KRT8*, *TFF3*, *VIL1*, *MUC2* and *MUC13* was upregulated in comparison to untreated control cells. The same experimental conditions in EPC2 cells provoked upregulation of columnar epithelium-specific *KRT7*, *KRT18* and *TFF3* mRNA as compared to untreated control cells.

Summarized alterations in BE-specific gene expression observed in human biopsies and in vitro and in vivo models were presented in Table 5.

**Figure 11:**
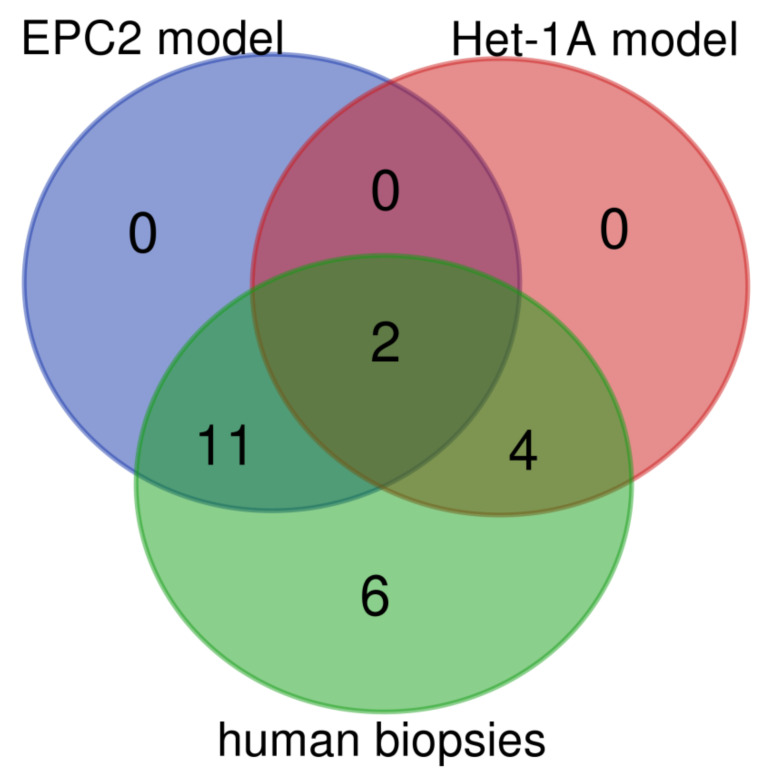
Venn diagram displaying numbers of up/downregulated genes in human biopsies derived from patients with Barrett’s metaplasia as compared with in vitro Het-1A and EPC2 model of Barrett’s esophagus. Overlap area shows the number of the same up/downregulated genes in the appropriate groups. Graphs were obtained through Venn diagrams software (available online: http://bioinformatics.psb.ugent.be/webtools/Venn/). Different colors mean different datasets.

Based on our data, EPC2 and Het-1A cells react differently to the implemented chronic mixed acid and bile treatment. Interestingly, when we took a deeper look into the outcome of our investigations in vitro, we found that the expression pattern of analyzed genes demonstrated around 57% of similarities between the human biopsies and the EPC2 cells treated with acidic BM (Figure 11, Table 5). In turn, in Het-1A model only 26% of genes reflected the expression pattern similar to that obtained in biopsies from BE patients (Figure 11, Table 5).

**Figure 12:**
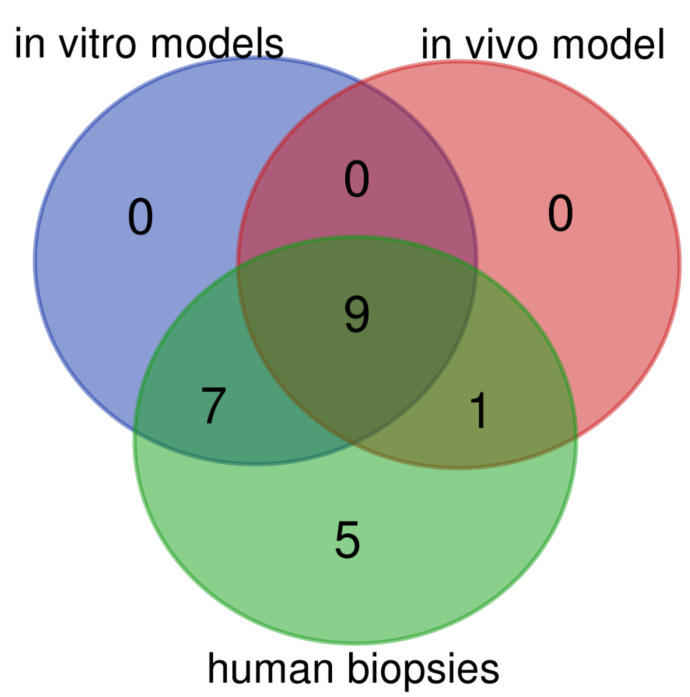
Venn diagram displaying numbers of up/downregulated genes in human biopsies derived from patients with Barrett’s metaplasia as compared with in vitro (Het-1A and EPC2) models and surgical animal model of Barrett’s esophagus. Overlap area shows the number of the same up/downregulated genes in the appropriate groups. Graphs were obtained through Venn diagrams software (available online: http://bioinformatics.psb.ugent.be/webtools/Venn/). Different colors mean different datasets.

Additionally, we have found that in animal BE model 45% of assessed genes were expressed in a pattern characteristic for human BE metaplasia (Figure 12, Table 5). In turn, when expression profile of both cell lines within the optimized in vitro BE model were analyzed together, 73% of genes reflected alterations observed in human biopsies (Figure 12, Table 5).

Taken together, we conclude that our optimized in vitro model based on two primary immortalized human esophageal squamous cell lines is suitable to observe an efficient induction marker specific for human BE epithelium. However, it is worth mentioning that cell cultures apparently lack systemic inflammatory response, the influence of esophageal microcirculation, microenvironmental factors, neural components, neuropeptides and cellular interactions characteristic for esophageal cells functioning in vivo [32,39]. Therefore, these doubts could be, at least in part, solved by studies in animal models of BE in vivo providing additional information about macroscopic, microscopic, functional and biochemical alterations. The animal model, applied simultaneously with the optimized in vitro model, could offer the opportunity for the evaluation of the molecular response and the effectiveness of possible drugs candidates targeting BE prevention and/or treatment.

## 4. Material and Methods

### 4.1. Analysis of BE Expression Profile for Selected Genes in Human Biopsies Based on GSE Datasets

Expression profiles from human biopsies derived from patients with normal esophageal epithelium and with diagnosed BE epithelium were obtained from Gene Expression Omnibus datasets GSE13083 (7 patients with normal vs. 7 patients with BE) [8], GSE34619 (8 patients with normal vs. 10 patients with BE) [9] and GSE1420 (8 patients with normal vs. 8 patients with BE) [24]. The results demonstrated on Table 1 are shown based on the analysis of the part of the data derived from the previously published databases [8,9,24]. Analyses were performed in silico using the NCBI Gene Expression Omnibus (GEO) database and the Gene Expression Omnibus 2R (GEO2R) tool (www.ncbi.nlm.nih.gov/geo/geo2r/). The results were represented as a log2-fold change (logFC) in BE samples vs. normal esophageal epithelium. For each logFC, an empirical Bayes moderated t-statistic was calculated by the software. Adjusted *p*-values, corrected for multiple testing using the Benjamini and Hochberg false discovery rate method, were taken for results interpretation. *p* < 0.05 was interpreted as statistically significant and marked in the table with an asterisk (*) for the genes with logFC values higher than 2 or lower than −2, which was considered as biologically significant up- or downregulation, respectively. Additionally, we further analyzed genes that were included in all three GSE datasets and were significantly up-/downregulated in at least one database.

### 4.2. Cell Cultures

The human SV40-immortalized esophageal squamous (Het-1A) epithelial cell line was a gift from J. W. P. M. van Baal (Utrecht University, Utrecht, The Netherlands). Het-1A cells were cultured in serum-free EPM2 medium (AthenaES, Baltimore, MD, USA). Het-1A cells were grown on FNC Coating Mix^®^ (AthenaES, Baltimore, MD, USA) containing fibronectin, collagen and albumin. The primary human telomerase reverse transcriptase (hTERT) immortalized esophageal epithelial (EPC2) cell line was a gift from K. K. Krishnadath (University of Amsterdam, Amsterdam, The Netherlands). EPC2 cells were cultured in keratinocyte-SFM (Life Technologies, Paisley, UK) medium supplemented with 50 μg/mL bovine pituitary extract (BPE) (Life Technologies, Paisley, UK) and 1.0 ng/mL human recombinant epidermal growth factor (EGF) (Life Technologies, Paisley, UK). Both culture media were supplemented with 100 U/mL penicillin and 50 mg/mL streptomycin (Sigma-Aldrich, Saint Louis, MO, USA). Cells were maintained at 37 °C and 5% CO_2_ and detached from the flasks prior to subculturing by the removal of the medium and the addition of 0.25% trypsin (Sigma-Aldrich, Saint Louis, MO, USA) for 1 to 5 min. These cell lines were selected as the most appropriate to be tested in the experimental model of BE, as described previously [1,32,38].

### 4.3. Acid/Bile Mixture (BM) Treatment

Het-1A and EPC2 epithelial cell lines were used to reflect the response of normal human esophageal epithelium to low pH and/or BM exposure. Both cell lines were seeded at a density of 10^5^ cells/well in 6-well plates. The cells were cultured until they reached approximately 40–50% confluence. At this stage, the cells were subjected to 1, 3 and 6 days of acid and/or BM treatment, with a 30-min period of exposure per day. The BM contained 25% deoxycholic acid, 45% sodium glycocholate hydrate and 30% sodium taurochenodeoxycholate (Sigma-Aldrich, Saint Louis, MO, USA); total BM concentration used in final experiments was 50 and 100 µM. The acidified medium consisted of appropriate culture medium adjusted to pH 5.0 in which pH was adjusted with 5 M HCl. Cells were also cultured in regular medium (pH 7.3 for EPM2 medium; pH 7.2 for keratinocyte-SFM medium) with/or without co-incubation with BM. After acid/BM exposure, the cells were rinsed with PBS, and then regular medium was added. After the last day of exposure, cells were left for 24 h and then lysed for RNA extraction. Cells were approximately 90% confluent at this time. The type and the molar ratio of bile salts in the BM have been based on studies analyzing gastroesophageal refluxate of patients with erosive esophagitis and BE [40,41]. Daily exposure time to BM and BM concentrations were selected based on cell viability assay data, cell morphology observations and previously published data [40].

### 4.4. Cell-Viability Assays

Cell viability was evaluated using thiazolyl blue tetrazolium bromide (MTT) colorimetric assay (Sigma-Aldrich, St. Louis, MO, USA). Het-1A and EPC2 cells were plated in 5 replicates in 96-well plates at a density of 10^4^ cells/well in a final volume of 100 μL medium. After overnight incubation at 37 °C, 5% CO_2_, dilutions of BM in acidified (pH 5.0) or regular medium were added in 5 replicates for 30 min. Untreated cells (appropriate volumes of medium added) served as controls. After 24 h, 50 μL of the MTT solution was added to each well and incubated for 4 h at 37 °C. Medium was removed and the formazan product of MTT reduction was dissolved in 75 μL of DMSO per well. The optical density was measured at 550 nm.

### 4.5. Analysis of mRNA Expression by Real-Time Polymerase Chain Reaction (PCR)

Total RNA was isolated using commercially available kit with spin-columns (Universal RNA/miRNA Purification Kit, EURx, Gdansk, Poland), according to the manufacturer’s protocol. RNA concentration was measured using Qubit 4 fluorometer (Thermo Fisher Scientific, Waltham, MA, USA). Reversed transcription to cDNA was performed using the High-Capacity cDNA Reverse Transcription Kit (Applied Biosystems, Foster City, CA, USA), using 1,8 µg of RNA for each reaction well.

Relative gene expression was determined by real-time PCR according to the MIQE guidelines. All reactions were performed in 96-well reaction plates in duplicates or triplicates via the Quant Studio 3 system (Applied Biosystems, Foster City, CA, USA). The 2X TaqMan Fast Advanced Master Mix (Thermo Fisher Scientific, Waltham, MA, USA) and 20X TaqMan gene expression assays (Thermo Fisher Scientific, Waltham, MA, USA) were used according to the manufacturer’s protocol (see gene IDs in Supplementary Materials Tables S1 and S2). PCR reaction conditions were as follows: (i) an initial incubation at 50 °C for 2 min, (ii) denaturation at 95 °C for 2 min, (iii) 40 cycles of 95 °C for 1 sec and 60 °C for 20 s. The relative quantitation of gene expression was performed using the 2^−ΔΔCT^ method with cDNA derived from untreated cells or physiological esophageal epithelium of rat as reference samples. *p* < 0.05 was interpreted as statistically significant for at least a two-fold up/downregulation in relative expression, which was considered as biologically relevant. Barrett’s-like samples were selected in the number of 5 for gene expression analysis in animal biopsies.

### 4.6. Animal Model of BE

The study was approved by the I Local Animal Care and Use Ethical Committee held by Jagiellonian University Medical College in Cracow and was run in compliance with the European Union regulations, ARRIVE guidelines and with implications for replacement, refinement or reduction (the 3Rs) principles, regarding handling of experimental animals (approval no 89/2017, permission date: 22 November 2017 and approval no 23/2016, permission date: 20 July 2016).

Male Wistar rats (*Rattus norvegicus*) in the total number of 15 were used in the experiments. Animals were fasted for 24 h before surgery with free access to drinking water. An anastomosis between the GEJ and the duodenum (esophagogastroduodenal anastomosis, EGDA) on its anterior mesenteric border was created to induce mixed duodenogastroesophageal reflux according to the method introduced by Nishijima et al. [30] and based on the generation of a shortcut for the chronic mixed gastroduodenal contents reflux through the damaged lower esophageal sphincter [15]. This surgical model with slight modifications has been widely described in scientific literature [33,42]. Briefly, under general isoflurane (2–4%) anesthesia, a midline laparotomy was performed and followed by a longitudinal incision extending approximately 5 mm along the lower part of the anterior esophagus wall, including the GEJ area. Next, the second incision of 5 mm in length was generated 4 cm distally from the Treitz ligament on the anterior mesenteric border of the duodenum. These incisions were side to side anastomosed using 7–0 silk sutures. The abdomen muscles and skin were closed separately with 4–0 silk sutures. After the surgical procedure and during the recovery phase, rats were infused s.c. with 5–10 mL of isotonic sodium chloride. For the next 10 weeks, the animals were fed a standard diet with free access to the drinking water. After that period, animals were sacrificed by i.p. administration of a lethal dose of pentobarbital (Biowet, Pulawy, Poland).

The esophagus and stomach were removed and opened longitudinally for macroscopic examination. For microscopic evaluation biopsies containing the esophagus, the GEJ and forestomach were sectioned. These segments were embedded in paraffin, cut into 4 μm sections and stained by haematoxylin/eosin (H&E) and alcian blue/periodic acid-Schiff (AB/PAS) for microscopic evaluation. Samples were evaluated using a light microscope (AxioVert A1, Carl Zeiss, Oberkochen, Germany). Digital documentation of histological slides was obtained using the abovementioned microscope equipped with automatic scanning table and ZEN Pro 2.3 software (Carl Zeiss, Oberkochen, Germany) to collect multiple photographs of each histological sample and to stitch them into one picture; to obtain better quality of each picture, the background was subtracted and unified as white [43]. Esophageal mucosal samples were collected for biochemical and molecular assessments on ice, snap-frozen in liquid nitrogen and stored at −80 °C until further analysis [43]. Blood samples were collected from the vena cava and serum was stored at −80 °C until further analysis [43].

The macroscopic degree of the esophageal mucosa injury and disease progression was assessed based on following criteria (lesion score):

0—physiological normal esophageal mucosa with squamous epithelium,

1—inflammation without ulcers reaching up to 1.5 cm of the esophagus as measured from GEJ,

2—inflammation without ulcers reaching beyond 1.5 cm of the esophagus as measured from GEJ,

3—inflammation with macroscopic ulceration and papillomatosis of the esophageal mucosa surface reaching up to 1.5 cm of the esophagus as measured from GEJ,

4—inflammation with macroscopic ulceration and papillomatosis of the esophageal mucosa surface reaching beyond 1.5 cm of the esophagus as measured from GEJ.

Presence or absence of the following criteria was included in the microscopic and histological analysis of the disease progression within esophageal mucosa:

1. hyperplasia of squamous epithelium,
2. fibrosis of lamina propria,
3. esophagitis: 1—thickening of squamous epithelium with basal cell layer occupying up to 30% of its height; elongation of connective tissue papillae, 2—regeneration layer occupying 50% of the epithelium thickness; hyperemia and scanty inflammatory infiltrate are present in connective tissue papillae, 3—expansion of the regeneration zone to 75% of the epithelial height; moderate inflammatory infiltrate in connective tissue papillae, 4—ulceration or massive inflammatory infiltrate,
4. Barrett’s-like lesion with the presence of goblet cells.

### 4.7. Determination of Serum Content of Pro- and Anti-Inflammatory Factors by Luminex Microbeads Fluorescent Assays

Serum concentration of IL-1β, IL-2, IL-4, IL-5, IL-6, IL-10, IL-12, IL-13, TNF-α, IFN-γ, GM-CSF was assessed using the Luminex microbeads fluorescent assays (Bio-Rad, Hercules, CA, USA) and Luminex MAGPIX System (Luminex Corp., Austin, TX, USA). Results were calculated from the calibration curves and expressed in pg/mL, according to the manufacturer’s protocol, as described previously [43].

### 4.8. Statistical Analysis

Analyses were performed using GraphPad Prism 5 (GraphPad Prism Software Inc., San Diego, CA, USA). Results are presented as mean ± SEM. Statistical analysis was performed with Student’s *t*-test or ANOVA with Dunnett’s multiple comparison if more than two experimental groups were compared. For all statistical analyses, the level of significance was set as *p* < 0.05.

## Figures and Tables

**Table 1:** Alterations in selected genes expression in human biopsies derived from patients with Barrett’s metaplasia as compared with normal squamous epithelium, based on analysis of the database no GSE13083 [8], GSE34619 [9] and GSE1420 [24]. Asterisk (*) indicates statistically significant difference with *p* < 0.05 in parallel with logFC values lower than −2 or higher than 2.

		Database No GSE13083 (*n* = 7): Barrett’s Metaplasia (7 Samples) vs. Normal Squamous Epithelium (7 Samples)			Database No GSE34619 (*n* = 18): Barrett’s Metaplasia (*n* =10) vs. Normal Squamous Epithelium (*n* = 8)			Database No GSE1420 (*n* = 16): Barrett’s Metaplasia (*n* = 8) vs. Normal Squamous Epithelium (*n* = 8)		
**Gene Symbol**	**Predicted Type of Epithelium**	**Gene ID**	**logFC**	***p* Value**	**Gene ID**	**logFC**	***p* Value**	**Gene ID**	**logFC**	***p* Value**
*KRT1*	squamous (esophageal)	205900_at	−7.49126	1.11e−05 *	7963491	−3.3024845	1.10e−03 *	205900_at	−2.217323	0.277797
*KRT4*	squamous (esophageal)	213240_s_at	−4.6401743	5.80e−02	7963534	−5.428193	2.16e−05 *	214399_s_at	0.9627934	0.291337
*KRT5*	squamous (esophageal)	201820_at	−6.9473657	1.02e−02 *	7963427	−5.19733	1.27e−05 *	201820_at	0.2659335	0.939807
*KRT6A, 6B, 6C*	squamous (esophageal)	214580_x_at	−5.0172743	3.66e−02 *	7963410	−4.5273685	7.31e−05 *	214580_x_at	0.3930203	0.885997
*KRT10*	squamous (esophageal)	207023_x_at	−1.9265443	4.80e−03 *	8015104	−1.9039765	1.43e−02 *	207023_x_at	0.0731168	0.925068
*KRT13*	squamous (esophageal)	207935_s_at	−5.3344986	4.04e−02 *	8015323	−5.5419725	5.55e−05 *	207935_s_at	0.4701058	0.861817
*KRT14*	squamous (esophageal)	209351_at	−4.0982471	1.17e−01	8015366	−2.2296553	1.88e−03 *	209351_at	1.928972	0.499067
*KRT15*	squamous (esophageal)	204734_at	−6.05938	2.09e−03 *	8015337	−4.686177	2.79e−10 *	204734_at	−0.327417	0.941066
*KRT16*	squamous (esophageal)	209800_at	−5.0731586	5.63e−03 *	8015376	−3.5205307	2.61e−05 *	209800_at	1.16582	0.682918
*KRT17*	squamous (esophageal)	212236_x_at	−2.6745814	8.21e−02	8005449	−1.7824035	1.42e−03 *	205157_s_at	2.6061311	0.297826
*KRT23*	squamous (esophageal)	218963_s_at	−2.0889186	3.74e−02 *	8015133	−1.2800192	7.70e−02	218963_s_at	0.0060589	0.997257
*KRT24*	squamous (esophageal)	220267_at	−4.4806771	2.26e−03 *	8015060	−2.905637	7.73e−05 *	220267_at	−0.676433	0.81305
*KRT7*	columnar (intestinal)	209016_s_at	2.2155471	4.07e−02 *	7955613	1.9065172	5.46e−06 *	209016_s_at	1.6998427	0.204673
*KRT8*	columnar (intestinal)	209008_x_at	6.4172871	2.18e−09 *	7963567	4.0091988	3.50e−11 *	209008_x_at	2.6806701	0.027973 *
*KRT18*	columnar (intestinal)	201596_x_at	3.4490243	5.03e−06 *	8154725	2.024285	2.16e−06 *	201596_x_at	1.9282774	0.082523
*KRT19*	columnar (intestinal)	201650_at	1.7536714	1.01e−03 *	8015349	0.759105	3.08e−02	201650_at	1.2141959	0.658343
*KRT20*	columnar (intestinal)	213953_at	8.5259443	3.44e−09 *	8015124	4.374532	2.00e−04 *	213953_at	4.8072071	0.037302 *
*TFF1*	columnar (intestinal)	205009_at	8.2017486	1.75e−05 *	8070579	6.4595337	1.46e−12 *	205009_at	5.6843253	0.010407 *
*TFF2*	columnar (intestinal)	214476_at	7.6665729	4.86e−05 *	8070574	5.2354248	1.23e−08 *	214476_at	5.8714482	0.006909 *
*TFF3*	columnar (intestinal)	204623_at	8.9897857	9.11e−08 *	8070567	2.2346355	2.20e−04 *	204623_at	3.3738183	0.110978
*VIL1*	columnar (intestinal)	209950_s_at	5.8340643	8.63e−09 *	8078665	2.2711873	1.88e−08 *	209950_s_at	2.5215122	0.025483 *
*MUC1*	columnar (intestinal)	213693_s_at	1.5493643	1.21e−01	7920642	1.9449625	1.69e−05 *	213693_s_at	1.1435938	0.427828
*MUC2*	columnar (intestinal)	204673_at	6.7226214	3.16e−06 *	7937560	2.1981597	2.77e−03 *	204673_at	2.9508572	0.271622
*MUC3A/B*	columnar (intestinal)	217117_x_at	0.7757457	9.80e−02	8135015	4.3127432	6.68e−09 *	217117_x_at	0.9212935	0.225325
*MUC4*	columnar (intestinal)	217109_at	−1.07354	1.13e−01	8092978	0.1271057	8.92e−01	204895_x_at	1.903752	0.360547
*MUC5ac*	columnar (intestinal)	214385_s_at	8.1766871	8.25e−07 *	not included in the database			214385_s_at	7.1466461	0.005318 *
*MUC5B*	columnar (intestinal)	213432_at	2.7593857	4.75e−02 *	7937612	1.6075105	2.00e−03 *	213432_at	2.3107749	0.510519
*MUC6*	columnar (intestinal)	214133_at	3.1026371	7.45e−03 *	7945595	5.9384813	1.46e−11 *	214133_at	3.4098322	0.017934 *
*MUC12*	columnar (intestinal)	not included in the database			8135033	2.2172345	1.33e−03 *	not included in the database		
*MUC13*	columnar (intestinal)	218687_s_at	7.3541829	5.65e−10 *	8090180	7.3474513	1.87e−11 *	218687_s_at	3.6273263	0.031468 *
*MUC15*	columnar (intestinal)	not included in the database			7947156	−4.7653307	4.28e−10 *	not included in the database		
*MUC17*	columnar (intestinal)	not included in the database			8135048	5.953779	6.32e−08 *	not included in the database		
*MUC21*	columnar (intestinal)	not included in the database			8177931	−5.732196	5.46e−07 *	not included in the database		

**Table 2:** Incidence of the particular macroscopic lesion score in esophageal mucosa of rats 10 weeks after esophagogastroduodenal anastomosis (EGDA)-inducing surgery.

Macroscopic Lesion Score	Number of Animals with EGDA (%) (*n* = 10)
2	1 (10%)
3	7 (70%)
4	2 (20%)

**Table 3:** Incidence of the selected microscopic criteria in esophageal mucosa of rats 10 weeks after esophagogastroduodenal anastomosis (EGDA)-inducing surgery.

Assessed Microscopic Criteria	Number of Animals with EGDA with Presence of the Criteria (%) (*n* = 10)
hyperplasia of squamous epithelium	10 (100%)
fibrosis of lamina propria	10 (100%)
Barrett’s metaplasia	6 (60%)
esophagitis with ulceration	8 (80%)

**Table 4:** Major differences between human and rat esophagus physiology and Barrett’s esophagus (BE) pathophysiology.

	Human	Rat
**Esophageal epithelium**	non-keratinized	keratinized
**Esophageal submucosal glands and papillae**	Present	Absent
**Stratum corneum**	Absent	Present
**Squamocolumnar transition at GEJ**	yes	no
**Natural reflux**	yes	no
**Natural BE to EAC progression**	yes	no
**Compartmentalized stomach (forestomach and distal stomach)**	no	yes
**BE progression time**	10 years	Around 2–3 months

**Table 5:** Summary of alterations in expression of squamous and columnar epithelium-specific genes observed in human biopsies and in vitro using Het-1A and EPC2 cells and in vivo EGDA rat model. A vertical up arrow (↑) indicates upregulation of mRNA expression in Barrett’s metaplasia as compared with samples without Barrett’s metaplasia/untreated control cells/intact rats (background in orange); a vertical down arrow (↓) indicates downregulation of mRNA expression in Barrett’s metaplasia as compared with samples without Barrett’s metaplasia/untreated control cells/intact rats (background in blue); a horizontal left right arrow (↔) indicates no changes in mRNA expression (background in dark grey); n.a. indicates no amplification; n.d. not determined (background in light grey).

Gene Symbol	Type of Epithelium	In Vitro Models		In Vivo Model	Human Biopsies
		Het-1A	EPC2		
*KRT1*	squamous	n.a.	↓	↑	↓
*KRT4*	squamous	n.a.	↓	↓	↓
*KRT5*	squamous	n.a.	↓	↑	↓
*KRT6*	squamous	↔	↓	n.d.	↓
*KRT13*	squamous	n.a.	↓	↓	↓
*KRT14*	squamous	n.a.	↓	↑	↓
*KRT15*	squamous	↓	↓	↓	↓
*KRT16*	squamous	↔	↓	n.a.	↓
*KRT23*	squamous	n.a.	↓	↔	↓
*KRT24*	squamous	n.a	↓	n.a.	↓
*KRT7*	columnar	↔	↑	↑	↑
*KRT8*	columnar	↑	↔	↑	↑
*KRT18*	columnar	↔	↑	↑	↑
*KRT20*	columnar	n.a.	n.a.	↑	↑
*TFF1*	columnar	n.a.	n.a.	↓	↑
*TFF2*	columnar	n.a.	n.a.	n.a.	↑
*TFF3*	columnar	↑	↑	↑	↑
*VIL1*	columnar	↑	n.a.	↔	↑
*MUC2*	columnar	↑	n.a.	↑	↑
*MUC3*	columnar	n.a.	n.a.	n.a.	↑
*MUC5B*	columnar	n.a.	n.a.	n.a.	
*MUC6*	columnar	n.a.	n.a.	n.a.	↑
*MUC13*	columnar	↑	↔	↑	↑

## Supplementary Materials

The following are available online at https://www.mdpi.com/1422-0067/21/17/6436/s1, Table S1: The list of selected human genes (and reference genes) and the corresponding TaqMan assays; Table S2: The list of selected rat genes (and reference genes) and the corresponding TaqMan assays.
