# Supplementary material for: Molecular Profile of Barrett’s Esophagus and Gastroesophageal Reflux Disease in the Development of Translational Physiological and Pharmacological Studies
Source: Int J Mol Sci. 2020 Sep 3;21(17):6436. doi: 10.3390/ijms21176436 (PMC7504401; doi:10.3390/ijms21176436)
Supplement: Supplementary file 1 [file ijms-21-06436-s001.pdf]

Table S1. The list of selected human genes (and reference genes) and the corresponding TaqMan assays.

No.	Gene name and symbol	Assay ID	NCBI gene reference
1	β-actin, ACTB	Hs99999903_m1	NM_001101.3
2	Glyceraldehyde-3-phosphate dehydrogenase, GAPDH	Hs99999905_m1	NM_002046.5
3	Keratin 1, *KRT1*	Hs00196158_m1	NM_006121.3
4	Keratin 4, *KRT4*	Hs00361611_m1	NM_002272.3
5	Keratin 5, *KRT5*	Hs00361185_m1	NM_000424.3
6	Keratin 6A, *KRT6A*	Hs01699178_g1	NM_005554.3
7	Keratin 7, *KRT7*	Hs00559840_m1	NM_005556.3
8	Keratin 8, *KRT8*	Hs01670053_m1	NM_001256282.1
9	Keratin 13, *KRT13*	Hs00999762_m1	NM_153490.2
10	Keratin 14, *KRT14*	Hs00265033_m1	NM_000526.4
11	Keratin 15, *KRT15*	Hs00267035_m1	NM_002275.3
12	Keratin 16, *KRT16*	Hs00373910_g1	NM_005557.3
13	Keratin 18, *KRT18*	Hs02827483_g1	NM_000224.2
14	Keratin 20, *KRT20*	Hs00300643_m1	NM_019010.2
15	Keratin 23, *KRT23*	Hs01119992_m1	NM_015515.4
16	Keratin 24, *KRT24*	Hs00962561_m1	NM_019016.2
17	Mucin 2, oligomeric mucus/gel-forming, *MUC2*	Hs00159374_m1	NM_002457.3
18	Mucin 3A/B, cell surface associated, *MUC3A/B*	Hs03649367_mH	NM_005960.1
19	Mucin 6, oligomeric mucus/gel-forming, *MUC6*	Hs01674026_g1	NM_005961.2
20	Mucin 5B, oligomeric mucus/gel-forming, *MUC5B*	Hs00861588_m1	NM_002458.2
21	Mucin 13, cell surface associated, *MUC13*	Hs00217230_m1	NM_033049.3
22	Trefoil factor 1, *TFF1*	Hs00170216_m1	NM_003225.2
23	Trefoil factor 2, *TFF2*	Hs00193719_m1	NM_005423.4
24	Trefoil factor 3, *TFF3*	Hs00173625_m1	NM_003226.3
25	Villin 1, *VIL1*	Hs00200229_m1	NM_007127.2

Table S2. The list of selected rat genes (and reference genes) and the corresponding TaqMan assays.

No.	Gene name and symbol	Assay ID	NCBI gene reference
1	β-actin, ACTB	Rn00667869_m1	NM_031144
2	Glyceraldehyde-3-phosphate dehydrogenase, GAPDH	Rn01462662_g1	NM_017008
3	Keratin 1, *KRT1*	Rn02346048_m1	NM_001008802
4	Keratin 4, *KRT4*	Rn02346072_m1	NM_001008806
5	Keratin 5, *KRT5*	Rn01533116_gH	NM_183333
6	Keratin 6, *KRT6*	not available	
7	Keratin 7, *KRT7*	Rn01533141_m1	XM_003750407
8	Keratin 8, *KRT8*	Rn01532759_g1	NM_199370
9	Keratin 13, *KRT13*	Rn01464231_m1	NM_001004021
10	Keratin 14, *KRT14*	Rn01467684_m1	NM_001008751
11	Keratin 15, *KRT15*	Rn01460389_m1	NM_001004022
12	Keratin 16, *KRT16*	Rn02345941_g1	NM_001008752

13	Keratin 18, *KRT18*	Rn01533362_g1	NM_053976
14	Keratin 20, *KRT20*	Rn00687576_m1	NM_173128
15	Keratin 23, *KRT23*	Rn01773106_m1	NM_001008753
16	Keratin 24, *KRT24*	Rn01471287_m1	NM_001004131
17	Mucin 2, oligomeric mucus/gel-forming, *MUC2*	Rn01498197_m1	XM_008760048
18	Mucin 3A, intestinal, *MUC3a*	Rn01481134_m1	XM_008769185
19	Mucin 5B, oligomeric mucus/gel-forming, *MUC5B*	Rn01502008_m1	XM_006230608
20	Mucin 6, oligomeric mucus/gel-forming, *MUC6*	Rn01759814_m1	XM_008760036
21	Mucin 13, cell surface associated, *MUC13*	Rn01647776_g1	XM_008768788
22	Trefoil factor 1, *TFF1*	Rn01428805_m1	NM_057129
23	Trefoil factor 2, *TFF2*	Rn00587721_m1	NM_053844
24	Trefoil factor 3, *TFF3*	Rn00564851_m1	NM_013042
25	Villin 1, *VIL1*	Rn01400773_g1	NM_001108224
